# Curing Mechanisms of Polymeric Nano-Copolymer Subgrade

**DOI:** 10.3390/ma16124316

**Published:** 2023-06-11

**Authors:** Shuang Shi, Miao Wang, Linhao Gu, Xiang Chen, Yanning Zhang

**Affiliations:** 1 School of Transportation, Southeast University, Southeast University Rd. #2, Nanjing 211189, China; shishuang@seu.edu.cn (S.S.); mobyzhangyn@163.com (Y.Z.); 2 China Road and Bridge Corporation, 88 Outer Andingmen Street, Beijing 100011, China; 3 School of Civil Engineering and Architecture, Nanjing Institute of Technology, 1# Hongjing Rd., Nanjing 211167, China; gu_linhao@163.com

**Keywords:** subgrade soil, polymeric nano-copolymer, curing mechanism, mechanical properties

## Abstract

The mechanical properties of the subgrade have a significant impact on the service life and pavement performance of the superstructure of pavement. By adding admixtures and via other means to strengthen the adhesion between soil particles, the strength and stiffness of the soil can be improved to ensure the long-term stability of pavement structures. In this study, a mixture of polymer particles and nanomaterials was used as a curing agent to examine the curing mechanism and mechanical properties of subgrade soil. Using microscopic experiments, the strengthening mechanism of solidified soil was analyzed with scanning electron microscopy (SEM), energy-dispersive spectroscopy (EDS), Fourier infrared spectroscopy (FTIR), and X-ray diffraction (XDR). The results showed that with the addition of the curing agent, small cementing substances on the surface of soil minerals filled the pores between minerals. At the same time, with an increase in the curing age, the colloidal particles in the soil increased, and some of them formed large aggregate structures that gradually covered the surface of the soil particles and minerals. By enhancing the cohesiveness and integrity between different particles, the overall structure of the soil became denser. Through pH tests, it was found that the age had a certain effect on the pH of solidified soil, but the effect was not obvious. Through the comparative analysis of elements in plain soil and solidified soil, it was found that no new chemical elements were produced in the solidified soil, indicating that the curing agent does not have negative impacts on the environment.

## 1. Introduction

A subgrade with excellent mechanical properties can enhance the service life and road performance of pavement superstructures. Over the years, scholars have strengthened the adhesion between soil particles by adding inorganic substances, polymers, and other admixtures, so as to improve the strength and stiffness of the soil and ensure the long-term stability of the pavement structure. At the same time, scholars have further studied the curing mechanism of different admixtures through multiscale means to optimize the mechanical properties of the roadbed.

Thompson M. R. [1] found that the curing rate of lime- and ash-solidified soils was much slower than that of cement-solidified soils, and the elastic modulus stress state (i.e., deviator stress and confining pressure) could be determined from the structural analysis of the test design (with due consideration of the upper layer and load pressure). Thompson also determined the relationship between the elastic modulus of solidified soil and the unconfined compressive strength (UCS). Puppala et al. [2] summarized the engineering characteristics of lime-solidified soil with different water contents from the aspects of the unconfined compressive strength, the California bearing ratio (CBR), and the elastic modulus compared with those of plain soil. The results showed that lime-solidified soil could improve the mechanical properties of raw materials, such as the modulus, but can also reduce the plastic properties and plastic strain of materials. Finally, the elastic modulus was analyzed by using the regression model with three constants. Mohammad et al. [3] found that under the same environmental conditions, the elastic modulus of lime- and cement-stabilized soils increased with the increase in curing agent content, while permanent deformation decreased with the increase in stabilizer content. Solanki et al. [4], in combination with AASHTO’s 2002 Mechanistic-Empirical Pavement Design Guide (MEPDG), analyzed material characteristics such as the resilience modulus (Mr), elastic modulus (ME), unconfined compressive strength (UCS), water sensitivity, etc., to determine the road properties of cement-based soil. Abu-Farsakh et al. [5] found that there was a strong log-linear relationship between the elastic modulus and the age of lime- and cement-stabilized soils. Consoli et al. [6] solidified silty clay and sandy clay by adding cement. They evaluated the solidified soil through strength tests and suction measurements by taking the mud content, porosity, porosity/cement ratio, and other indicators as influencing factors. The results showed that there was a certain fitting relationship between porosity and cement content and between porosity and strength. Taheri et al. [7] showed that deviatoric stress, confining pressure, and compaction delay time had an impact on the stiffness (or elastic modulus) of solidified soil and put forward an empirical formula.

In recent years, the triaxial test has become an important means to study soil materials in the laboratory because it better mimics the stress state of the soil in practical engineering. For nonlinear soil materials, most constitutive models are based on triaxial test results, so the dynamic triaxial test is also applied in the study and analysis of solidified soils. Liang et al. [8] studied the dynamic resilience modulus of soil cement and found that when the dynamic stress was greater than the critical dynamic stress, the cumulative strain and elastic strain rapidly increased with the increase in the loading duration of dynamic stress. The elastic modulus decreased with the increase in dynamic stress, and the elastic strain and elastic modulus linearly increased with the increase in dynamic stress. Yonghui Shang et al. [9] investigated cement-stabilized expansive soil as the core layer of a heavy-duty railway subgrade, where they analyzed the variation law of parameters such as the dynamic strength and the damping ratio of cement-stabilized expansive soil through a dynamic triaxial test. They compared the results with those of solidified ordinary expansive soil, lime-stabilized expansive soil, and coarse filler. The results showed that the modulus and average critical dynamic stress of cement-stabilized expansive soil were significantly increased compared with those of remolded ordinary expansive soil. Onyelowe [10] predicted the stiffness of nano palm-branch gray soil in stabilizing a laterite roadbed and found that the strength and stiffness of the solidified soil were significantly improved in this prediction. At the same time, a nonlinear multiple regression relationship was established, which could predict the stiffness of subgrades well. Wei et al. [11] used industrial waste fly ash (FA) and oil shale ash (OSA) to strengthen silty clay (SC), which can be used as a subsoil material. Through studying the static and dynamic mechanical properties of solidified soil under optimal ratio of OSA/FA/SC, the results showed that the addition of FA and OSA improved the physical properties of silty clay, and there was no pollution of second-class surface water or third-class groundwater.

Wang et al. [12] conducted cyclic triaxial tests for properties such as the soil compacting coefficient, water content, deviator stress, load frequency, etc.; it was found that train speeds should be limited during the wet season to reduce the deviated stress of the subgrades through a comparison of the test results of subgrade stress. Zou [13] obtained the laws between the stress–strain characteristics and strength of lime-solidified soil, between the stress–strain characteristics and cohesion of lime-solidified, and between the stress-strain characteristics and internal friction angle of lime-solidified soil through dynamic triaxial tests. Niu [14] used dynamic triaxial tests to analyze the characteristics of the variation in the dynamic elastic modulus of lime-remolded loess under different environmental and stress conditions, and they deduced the constitutive relationship of lime-solidified loess using R.L. Kondner’s hyperbolic model.

Kamon et al. [15] solidified soil by mixing industrial wastes with lime and evaluated the formation of and microstructure changes in the chemical reaction products in the solidified soil via XRD and SEM. The results showed that the new curing agent significantly improved the early compressive and flexural strength of the solidified soil. Cerato [16] mixed lime, C-grade fly ash, and cement kiln ash into different fine-grained soils as curing agents. The content of calcium oxide (CaO) stabilizer in several kinds of reinforced subgrade soil was determined via X-ray fluorescence (XRF). The results showed that XRF accurately determined the content of calcium oxide in the different soils, showing it is an effective tool for the quality control of road construction. Sudheer [17] studied the influence of cement kiln dust (CKD) and stabilizer (RBI81-grade) on expansive clay with high plasticity. The soil’s chemistry before and after treatment was studied via SEM and EDS. The results showed that CKD can reduce the maximum dry density and improve the soil density. After the treatment, the soil compression coefficient significantly decreased, and the SEM images clearly showed the formation of CSH and CAH gels. Sukmak [18] studied the strength and microstructure characteristics of soft soil stabilized with palm oil fly ash as a curing agent. XRD, SEM, EDS, and FTIR spectroscopy were used to analyze the formation of stable soil structure. Muhammad [19] used an alkaline activator and magnesium chloride (MgCl_2_) to activate silica and alumina components in silt and studied the microstructure of the solidified soil using FESEM, EDS, and FTIR. The results showed that the chemical additive significantly improved the compressive strength of the soil mass and produced a gelatinous gel during the stabilization process, consisting of magnesium silicate hydrate (M-S-H) and magnesium aluminate hydrate (M-A-H) compounds, which bonded the soil particles together. Sudhakaran [20] used areca fiber and fly ash (BA) in subgrade reinforcement soil and studied the mineralogy and microstructure of the stabilized soil samples via XRD and SEM. The results showed that the formation of gelling compounds was confirmed by the XRD pattern, and the development of a dense matrix was shown in the SEM images. Therefore, a soil subgrade mixed with BA and areca fiber can be used as a small-volume pavement material. Jha [21] defined the long-term strength behavior mechanism of lime-treated soil based on the changes in the ion exchange (Ca^2+^, Mg^2+^, Na^+^, K^+^), mineralogy, and microstructure and performed a series of physicochemical and microscopic analyses such as XRD, SEM, and EDAX. The results showed that lime had a great effect on the concentrations of Ca^2+^ and Na^+^ ions in soil.

Lu et al. [22] solidified the waste slag of limestone generated in the project and revealed that the reason why the waste slag of limestone remolded by cement was inferior to that remolded by lime through XRD, SEM, particle size analysis, and other tests was the content of calcium hydroxide in the soil. Cai et al. [23] analyzed and compared the freeze–thaw durability of active MgO carbonation and cement-solidified soil via indoor triaxial testing, and they used SEM for microscopic image analysis. The test showed that the micropores of the active MgO carbonation solidified soil significantly reduced, leading to slightly lower strength. Que [24] compared the effects of cement, lime, EN-1, and other curing agents on soil reinforcement of Fujian high-liquid-limit clay through mechanical property analysis, durability tests, microscopic tests, and numerical calculation. The results showed that the improvement effect of the ionic curing agent and polymer curing agent was obvious. The settlement of the embankment after solidification was more than 10 cm less than that of plain soil, and both met the requirements of standard filling construction.

Based on this literature review, many studies have been conducted on the macroscopic and microscopic characteristics of solidified soil. In this study, a hybrid material composed of polymer particles and nanomaterials was used as a stabilizer for subgrade soil. The curing mechanism of solidified soil was analyzed using scanning electron microscopy (SEM), Fourier transform infrared spectroscopy (FTIR), and X-ray diffraction (XRD), aiming to provide a new solution for subgrade soil solidification.

## 2. Materials and Methods

### 2.1. Materials

#### 2.1.1. Basic Properties of Subgrade Soil

In this study, the soil was taken from the construction area near Jiulong Lake Campus of Southeast University, and its air-dried moisture content was 9.6%. The particle composition, liquid plastic limit, optimum moisture content, maximum dry density, and other performance indices of soil were tested according to the relevant provisions of the Highway Geotechnical Test Regulations (JTGE40-2007). After screening the soil particles, the gradation was as shown in Table 1:

As soil has different characteristics for different water contents, the liquid–plastic limit is an important evaluation index of soil, and the gap between the liquid–plastic limit, which is the plastic index (PI), is often used as a characterization parameter. In this study, the soil sample was a low-liquid-limit clay, which was denoted as CL.

**Figure 1:**
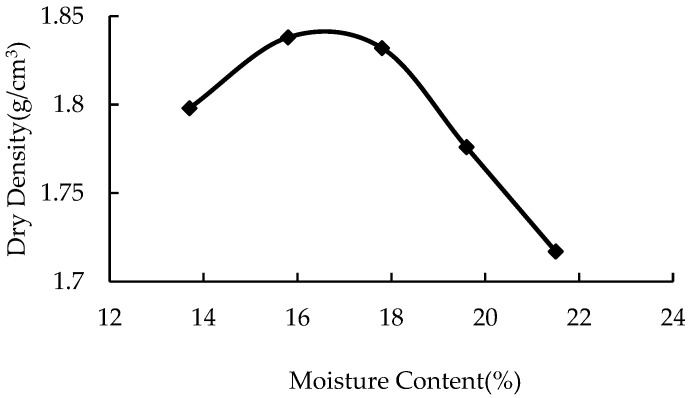
Dry density vs. moisture content.

The statistical results of the compaction test are shown in Table 2. According to the test results, the water content was taken as the horizontal coordinate and the corresponding dry density as the vertical coordinate. All points were connected, and the curve was fitted, as shown in Figure 1. According to the fitting curve in the figure, the optimal water content of plain soil was 16.3%, and the corresponding maximum dry density was 1.84 g/cm^3^.

#### 2.1.2. Basic Properties of Curing Agent

**Figure 2:**
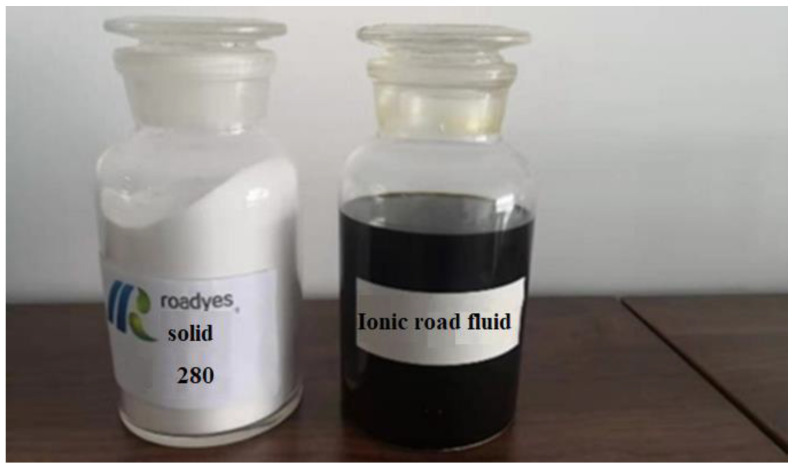
Curing agent.

The liquid road-curing agent used in this study (as shown in Figure 2) was a kind of new polymer curing agent, which was composed of binders and fillers. The binder was prepared from a polymer with high cohesion, and the filler was nano-Al_2_O_3_ particles.

This stable colloid was formed through the reaction of the road-curing agent with SiO_2_ and Ca(OH)_2_ in the soil; it could improve the compaction effect by eliminating the friction between soil particles and could enhance the ion connection between soil particles by penetrating the hydration film on the surface of soil particles, so as to ensure the strength and stiffness of the soil. Its technical indicators are shown in Table 3.

### 2.2. Characterization Methods

#### 2.2.1. SEM Test

The micro properties of soil are closely related to its macro performance. The evaluation of the curing mechanism and the effect of solidified soil should be considered in combination with the analysis of the microstructural properties of soil before and after curing. In this study, the pore and structural changes of soil before and after solidification were qualitatively analyzed via SEM to infer the curing mechanism of the curing agent.

For the preparation of sample blocks for scanning electron microscopy, standard cylindrical specimens with diameter × height (50 mm × 50 mm) were formed via static pressing first, and preserved until the duration of the curing reached the target age. The large specimen was broken into small pieces to ensure that one surface was not disturbed, and a sample of about 1 cm × 1 cm × 0.5 cm was removed from the test block with a blade.

**Figure 3:**
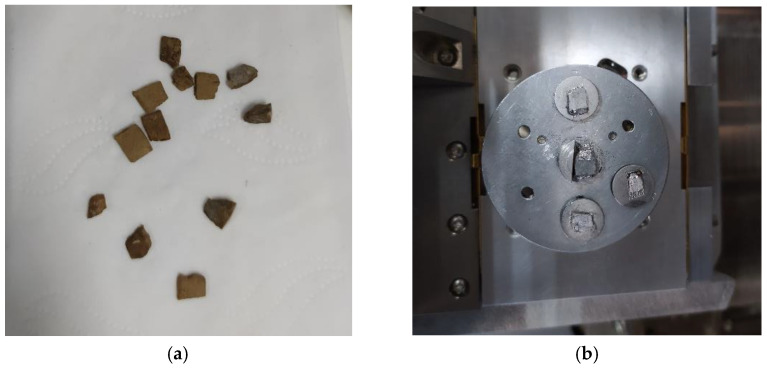
SEM sample preparation: (**a**) specimen preparation; (**b**) sample after vacuum and gold plating.

In order to reduce the observation error, sample drying was required for the use of scanning electron microscopy. In this study, the sample was air-dried for 2 d and kept under a vacuum for 2 d to keep it dry. Finally, the sample surface was gilded, as shown in Figure 3. The test was conducted with SEM (JEOL, JSM-7600F, Tokyo, Japan). It was concluded from the previous macro test that the optimal dosage of curing agent was 0.025%. Therefore, a comparative analysis was conducted between the plain soil sample and the solidified soil with curing agent dosages of 0.015% and 0.025% and a regimen age of 7 d. At the same time, for the solidified soil with a curing agent dosage of 0.025%, the changes in the microstructure under regimen ages of 7 days and 21 days were compared.

#### 2.2.2. pH-Value Examination

**Figure 4:**
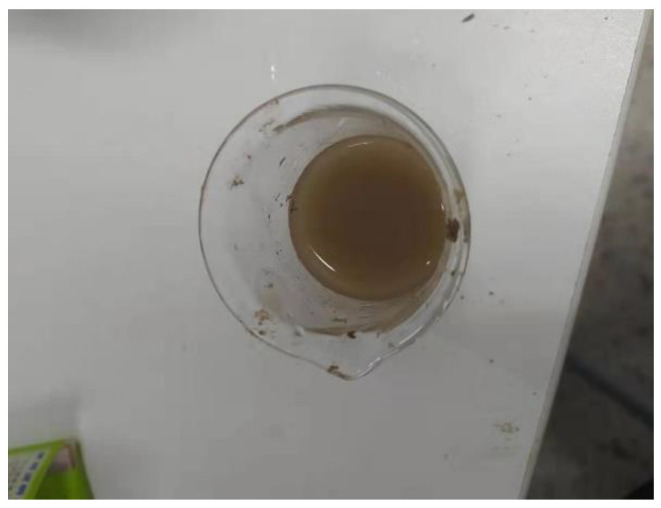
pH examination.

The pH value reflects the influence of the curing agent on the surrounding soil through the pH of the soil particles suspended in water. The changes in the pH value with time and dosage are important references for the comprehensive evaluation of the applicability of the curing agent. In this study, the pH value was measured for different-aged (3 d, 7 d, 14 d, 21 d, 28 d) solidified soil at a curing agent dosage of 0.025%. For the detailed procedure of the test, we followed the ASTM 4972 specifications, as shown in Figure 4.

#### 2.2.3. EDS Test

In the process of soil reinforcement, organic curing agents usually show a certain ion exchange effect. By changing the charged property of soil particles through ion reactions, it further changes the hydrophilicity of the soil and replace cations in the soil body, which can more closely combine the soils. During the curing process, a certain element change may occur. Thus, the element distribution in the dense structure observed via SEM in this study was analyzed for plain soil and solidified soil at 21 days of age and a 0.025% curing agent content through EDS (Apollo XLT SD, EDAX, Warrendale, USA).

#### 2.2.4. FTIR Test

In the process of soil solidification, the macromechanical properties and stability of the soil are improved to a certain extent, and the related properties of functional groups may affect the reaction between the curing agent and the soil, thus affecting the reinforcement effect. In this study, a mixture of polymer particles and nanomaterials was used as the curing agent, which had a certain number of functional groups.

**Figure 5:**
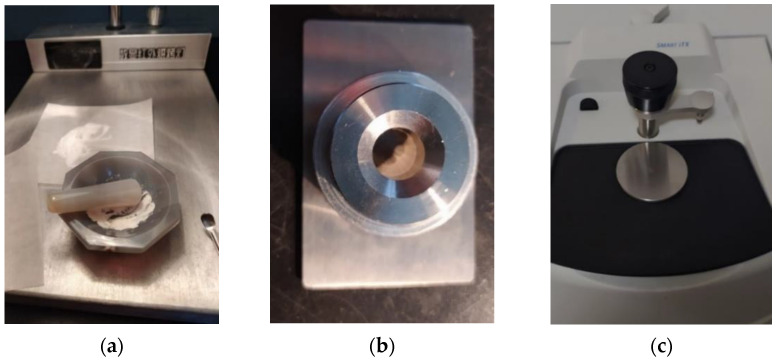
Fourier infrared spectroscopy (FTIR) test. (**a**) Potassium bromide mixed grinding. (**b**) KBr pellet. (**c**) ATR.

The functional group analysis was carried out for plain and solidified soil at 21 d of age and 0.025% curing agent dosage and for the curing agent stock solution. The infrared spectrum was detected with FTIR (NicoletiS10, Thermo Scientific, Waltham, USA, as shown in Figure 5.

#### 2.2.5. X-ray Diffraction Analysis

**Figure 6:**
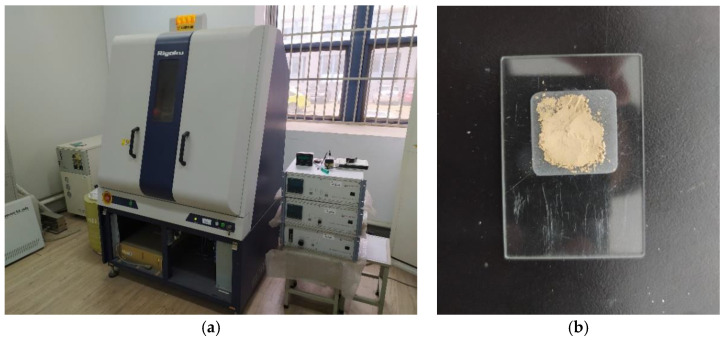
Smart X-ray diffractometer with sample preparation. (**a**) Intelligent X-ray diffractometer. (**b**) Observed specimen.

During the use of an organic curing agent, the changes in soil minerals are unknown. XRD (Ultima IV type, Rigaku, Japan) (Figure 6) is one of the important methods used to study the crystal structure, type, and the position distribution of particles. In this study, XRD was used to analyze the influence of the polymer curing agent on the mineral composition of the soil by comparing the mineral composition of solidified and plain soil. The preparation method of the XRD samples was the same as that for the infrared spectrum test. Cu target radiation was used in the test process. The measurement angle was set to 3° to 65°, the step length of the goniometer was set to 0.02°, and the measurement speed was set to 2°/min.

## 3. Results and Discussion

### 3.1. Microstructure Analysis of Solidified Soil

The microstructure characteristics of the subgrade soil have a significant impact on the macroscopic performance of a road. The particle size, shape, and arrangement of soil affect the macroscopic properties, such as moisture content, pore structure, and stability, which ultimately affect the stability and durability of the subgrade. In addition, modifying agents or polymer materials can alter the soil microstructure, improving its resistance to deformation and cracking. Analyzing and researching the microstructure of subgrade soil can provide a scientific basis for subgrade design and maintenance.

**Figure 7:**
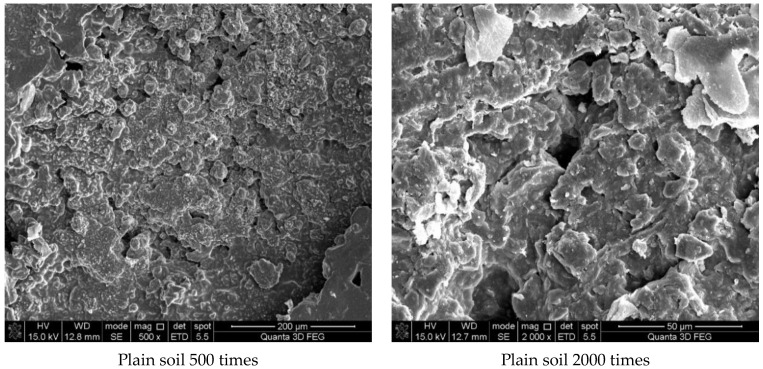

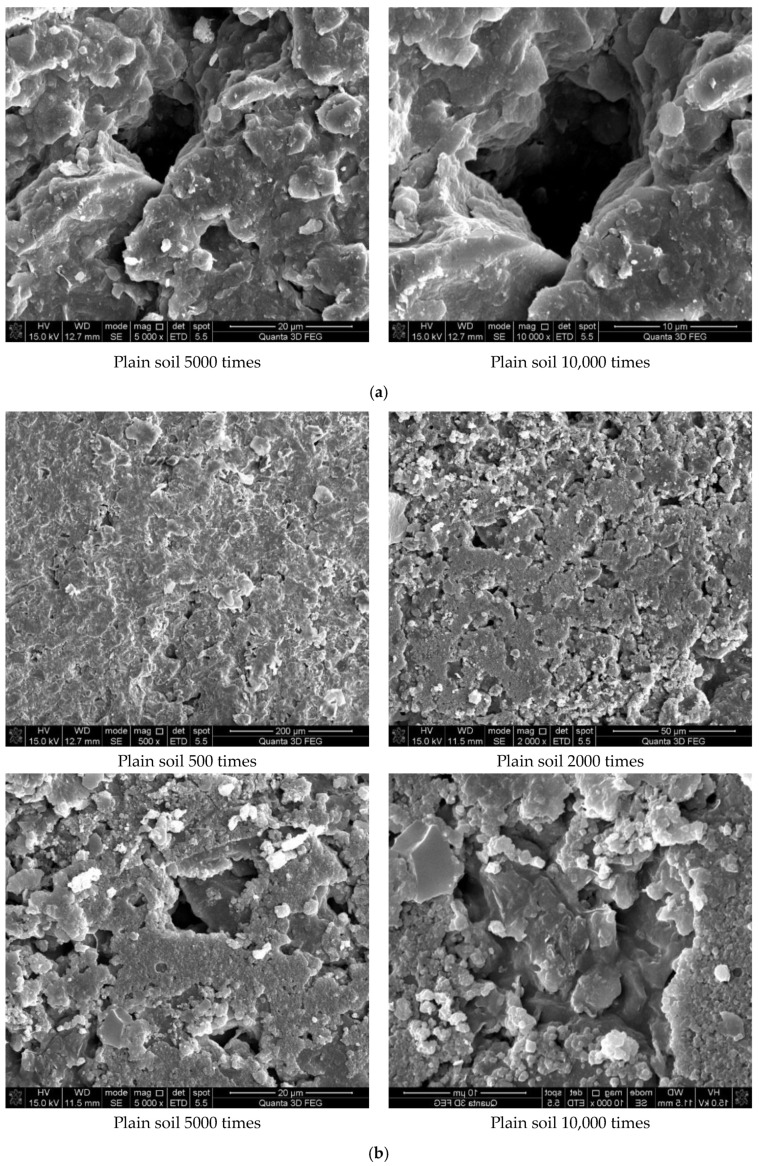

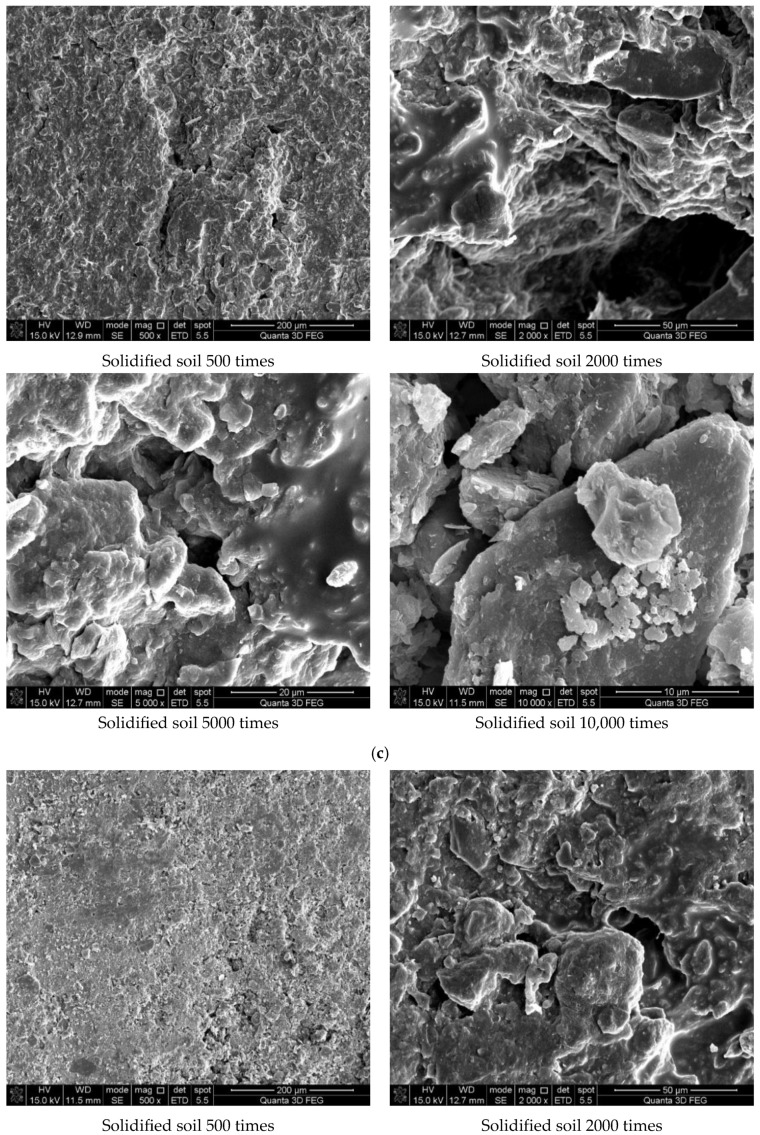

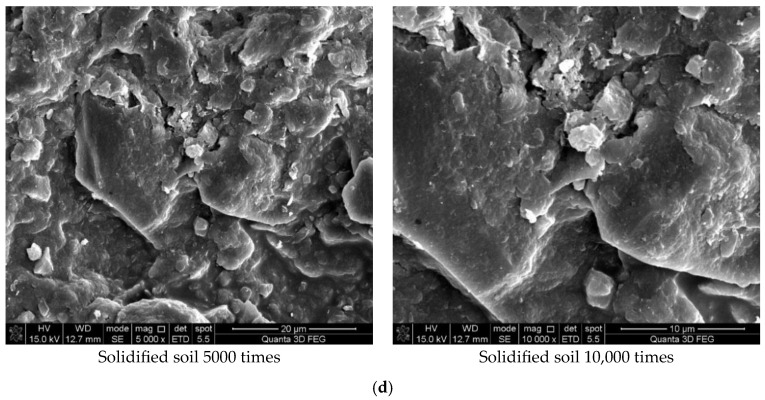
Scanning electron microscope test results. (**a**) Microstructure of plain soil. (**b**) Microstructure of solidified soil with 0.015% was preserved for 7 days. (**c**) Microstructure of solidified soil with 0.025% curing agent dosage was preserved for 7 days. (**d**) Microstructure of solidified soil with 0.025% curing agent dosage was preserved for 21 days.

Figure 7 shows the results of the experiment. It can be concluded from Figure 7a that the soil was mostly composed of free mineral particles and certain thin-layered units. All soil particles bonded in point-plane contact to form an overhead structure with many and large pores. As shown in Figure 7b, it was found that there were still many pores on the surface of the soil structure after adding the curing agent. However, after magnification, it could be seen that there were relatively obvious cementing substances on the surface of soil particle minerals, which made the surface of different minerals have a certain overall bond and reduced the porosity. At the same time, these colloidal particles filled the pores between the soil particles to a certain extent and played a certain role in bonding the soil particles. As shown in Figure 7c, the microstructure of the soil with 0.025% curing agent added was significantly tighter than that with 0.015% curing agent, and more cementing material formed on the mineral surface to fill the pores. At the same time, fine colloidal particles formed a certain agglomeration structure, which made the different mineral particles bond more closely and improved the integrity of the soil grain structure. Compared with Figure 7d, after 21 days of curing, a layer of relatively dense aggregate structure formed on the surface of soil particles, which connected the minerals in different positions. Therefore, it could be inferred that the curing agent reacted more fully with the minerals in the soil under a certain curing environment and curing age.

### 3.2. Analysis of pH Value Detection Results of Solidified Soil

**Figure 8:**
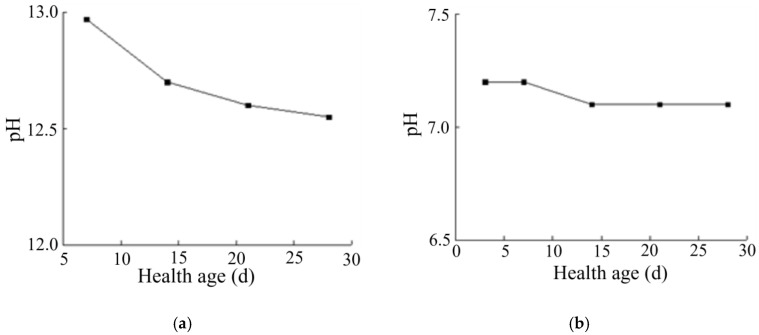
Variation in pH of solidified soil. (**a**) Variation in pH of solidified soil with age. (**b**) Variation in pH of cement-cured soil with age.

As can be seen from Figure 8a, with the increase in curing age, the pH of the solidified soil with a curing agent dosage of 0.025% reached a relatively stable value with relatively little change. This is because the curing agent was added after dilution, and the added content of curing agent proto solution was still very low. At the same time, the polymer and copolymer in the curing agent could react with some components in the soil, which changed the pH value of the soil to a certain extent. However, the polymer was wrapped in water and could not immediately react with the relevant components in the soil. With the increase in the curing age, the curing agent reacted with the minerals in the soil and formed the corresponding structure, which promoted the polymer and mineral particles to more closely combine and the reaction to more fully occur. In summary, after the soil sample was improved by the curing agent, the pH of the soil was about 7.1.

By comparing the changes in the pH of lime-solidified soil with age (see Figure 8b), we found that the pH change of lime-solidified soil decreased to a certain extent with the increase in curing age, which was not obvious in the solidified soil. Therefore, the specific reaction mechanism between lime-solidified soil and some substances needed to be determined through analysis of elements, functional groups, and minerals.

Any inorganic solidified soil, such as lime, greatly increases the pH value of the soil, which can reach 12 after the addition of curing agent, which can strongly impact the surrounding environment. The curing agent had no strong influence on the soil or the environment around the construction site because of the low dosage. One of the reasons could be the fact that the additive was a polymer, which was practically neutral and did not affect the amount of reacidification of the mixture.

### 3.3. Chemical Element Analysis of Solidified Soil

**Figure 9:**
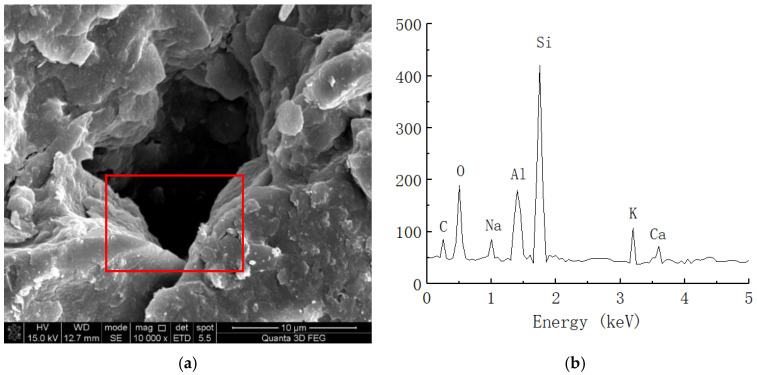
Spectral analysis of plain soil. (**a**) Microstructure of plain soil (10,000 times magnification). (**b**) Elemental analysis of plain soil.

**Figure 10:**
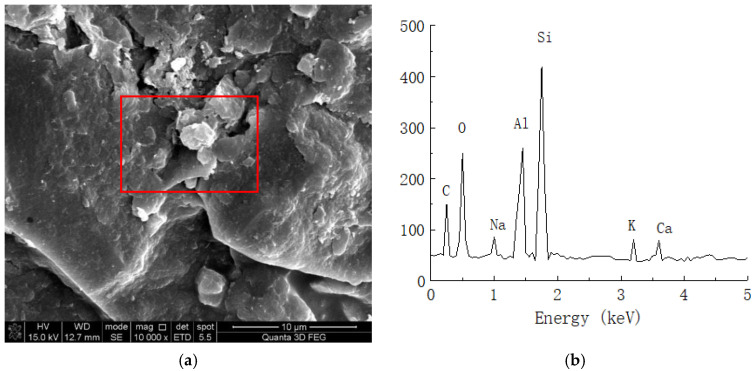
Spectral analysis of solidified soil. (**a**) Microstructure of solidified soil (10,000 times magnification). (**b**) Elemental analysis of solidified soil.

The energy spectrum analysis results of the plain and solidified soil are shown in Figure 9 and Figure 10, respectively.

As shown in Figure 9, the clay mainly contained C, O, Al, Si, K, Ca, and other chemical elements. Because soil mainly contains O, Al, Si, Ca, and other elements, we speculated that certain amounts of C and O are provided by impurities such as humus in the soil. Figure 10 shows the energy spectrum of the elements of the solidified soil with 0.025% curing agent content at 21 days. Compared with the plain soil, we found that the main elements of the solidified soil were still O, Al, Si, and Ca, but there were still some changes in the element contents of the solidified soil. On the one hand, the content of C in the soil increased to a certain extent, which reflected that the organic curing agent reacted with the minerals in the soil to form cementing substances, so the macromolecular chains of organic matter were also preserved in the soil. On the other hand, the content of Al ions significantly increased, which indicated that there was a considerable amount of Al^3+^ in the curing agent and chemical reaction with the minerals in the soil.

### 3.4. Functional Group Analysis of Solidified Soil

**Figure 11:**
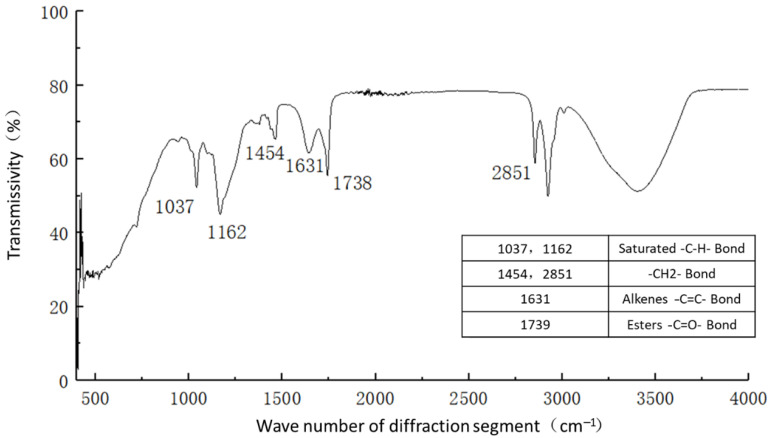
Functional group analysis of curing agent.

**Figure 12:**
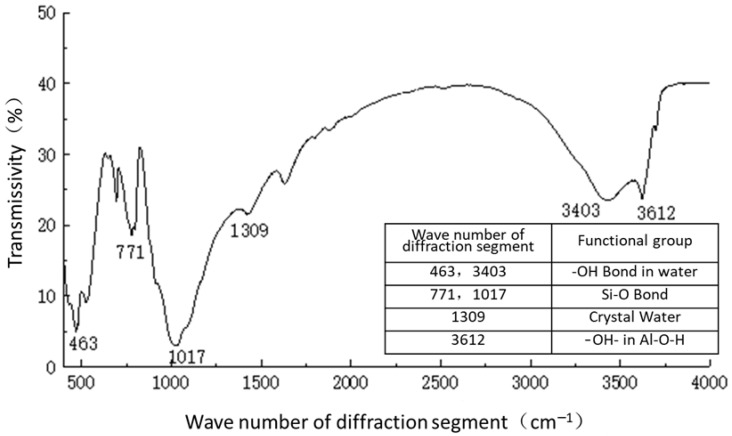
Functional group analysis of plain soil.

**Figure 13:**
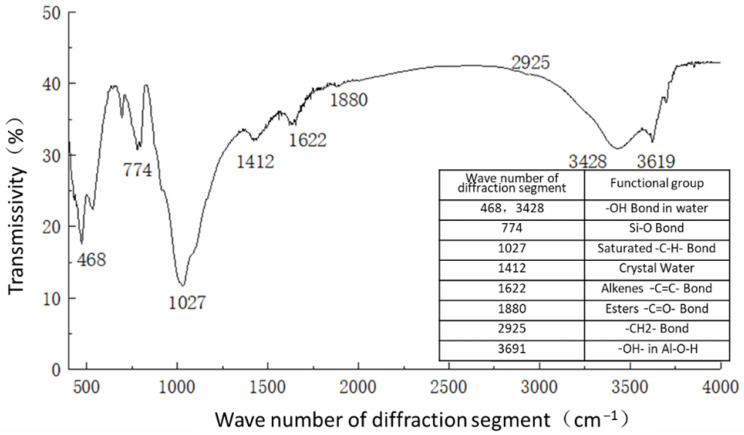
Functional group analysis of solidified soil.

For infrared spectrograms, when the transmittance is 10–80%, transmittance T is generally used as the ordinate for plotting. When the transmittance is greater than 80%, absorbance A is generally used as the ordinate for plotting. For the samples of plain soil, solidified soil, and curing agent, most of their transmittances were concentrated in the range of 10–80%, so the transmittances were taken as the ordinate, and the number of diffraction bands was taken as the horizontal coordinate. The spectral analysis results are shown in Figure 11, Figure 12 and Figure 13.

Figure 11 shows the functional groups of the curing agent. The curing agent was mainly composed of polymer and copolymer binder, including vinyl acetate ester-vinyl copolymer, nano-Al_2_O_3_ filler, reinforcing agent, organic ester dispersant, and other materials. According to the results of the Fourier infrared spectroscopy test, the curing agent mainly contained saturated -C-H bonds (1037, 1162 cm^−1^), -CH2- bonds (1454, 2851 cm^−1^), olefin -C=C- bonds (1631 cm^−1^), ester -C=O- bonds (1739 cm^−1^), and other active functional groups. The main functional groups of the curing agent were in the range of 1000–1800 cm^−1^.Figure 12 shows the analysis of the main active functional groups of the plain soil. The clay mainly contained -O-H bonds in water (463, 1309, 3403 cm^−1^), Si-O bonds in silicon dioxide (771, 1017 cm^−1^), Al-O-H bonds in Al_2_O_3_·3H_2_O (3612 cm^−1^), and other active functional groups. Figure 13 shows the main active functional groups of the solidified soil, by comparing the differences in the functional groups between the solidified soil, curing agent and plain soil, we found that no new functional group was generated during the curing process of the plain soil with curing agent, which indicated that the types of functional groups in the solidified soil were the same as those in the curing agent and plain soil. Additionally, the difference between the main functional groups of the solidified soil and the plain soil was in the 1000–2000 cm^−1^ diffraction band, which is consistent with the range of the main functional groups of the curing agent. Therefore, we judged that the polymer curing agent reacted with some components in the soil and formed a relatively stable and cohesive cementing substance, which existed in the clay minerals.

### 3.5. Mineral Composition Analysis of Soil Particles

**Figure 14:**
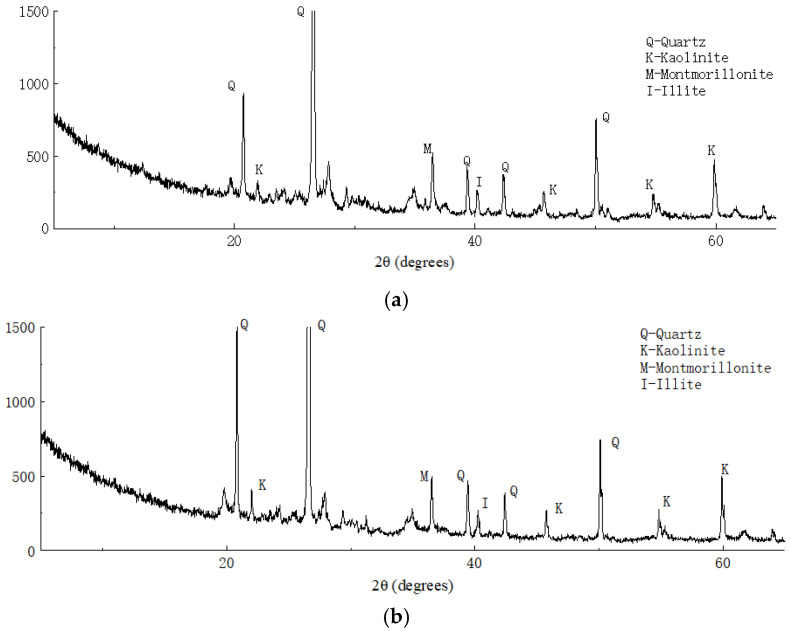
X-ray diffraction analysis results of (**a**) plain and (**b**) solidified soil.

Figure 14 shows the X-ray diffraction analysis results of the plain and solidified soil. Through comparison of the XRD data with those of common rock and soil minerals, we found that the main minerals in the clay were quartz, kaolinite, montmorillonite, and illite. Compared with the X-ray diffraction analysis results of the solidified soil, no new wave crests appeared in the results of solidified soil, which indicated that no new minerals were produced before and after soil solidification and ion exchange may have occurred between the curing agent and soil minerals.

Scherrer’s formula is commonly used in soil mineral analysis to analyze the subcrystalline size of minerals, and its expression is shown in (1):

(1) $$D=\frac{K\gamma }{\beta \cos\theta }$$

where D is the average thickness of the subcrystalline dimensions perpendicular to the direction of the crystal plane (nm); β is the sample diffraction peak half-peak width (rad); K is the Scherrer constant, which has a value of 0.89; λ is the X-ray wavelength, the value of which is 0.154056 nm; θ is the Bragg diffraction angle (degrees).

Table 4 shows a comparative analysis of the subcrystalline sizes of the main minerals in the soil samples before and after solidification. As shown in Table 4, the subcrystalline sizes of quartz, kaolinite, illite, and other major minerals reduced to varying degrees. It was found that the polymer contained in the curing agent reduced the subcrystalline size of the minerals and increased the density of soil by changing the adsorption type of the soil surface ions, which means that the high-valence cations were replaced by low-valence cations with a large radius; furthermore, the surface charge size and zeta potential of the minerals changed.

### 3.6. Microscopic Analysis of Strength-Formation Mechanism of Solidified Soil 

The organic curing agents are more easily combined with each other when the properties of the particles of the soil are changed, which further improves the strength and durability of the soil. The curing agent used in this study was mainly a polymer-based composite, which produced ion substitution and gelling effects.

Firstly, it was found that the curing agent mainly contained olefin -C=C- bonds, ester -C=O- bonds, and other active functional groups. The functional groups contained in the solidified soil included the same functional group types as the plain soil and curing agent, which indicated that the polymer and copolymer underwent a chemical reaction with some of the minerals or salts in the soil. The reaction further generated certain cemented substances and clays to achieve a better combination with the original minerals in the soil. By analyzing the chemical composition of the main component in the curing agent (vinyl acetate-vinyl copolymer) and referring to the curing principle of inorganic curing agents and the element analysis of the curing soil via EDS, we found that the most important chemical reaction was the reaction of curing agent with SiO_2_ and Ca(OH)_2_ in the soil to produce calcium silicate hydrate (C-S-H gel). The specific reactions were as follows:


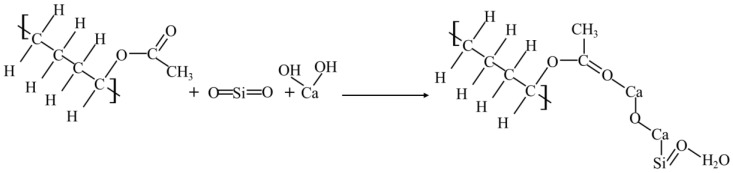


Because of the Al_2_O_3_ in the curing agent filler, it could also react with the minerals in the soil, generating a more stable substance than calcium silicate hydrate-aluminum oxide hydrate colloid (Al_2_O_3_·3H_2_O). Its chemical reaction is as follows:


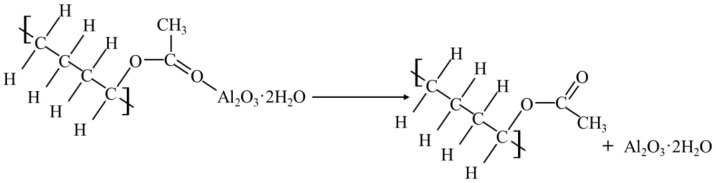


It could be seen that the particles formed by the reaction between the curing agent and the soil were filled in the pores of the soil, which made the soil particles combine more closely enhanced the compaction degree of the soil; and reduced the porosity to a certain extent. At the same time, the colloidal particles of these macromolecular chains also combined with the minerals in the soil to form the corresponding structure, which made the chemical composition and structure of the soil more stable and formed a cohesive structure, which enhanced the strength and stiffness of the solidified soil.

Secondly, from the XRD analysis of soil mineral surface elements, it was found that the curing agent changed the adsorption type of the soil-surface ions. The crystal size of the mineral obviously decreased before and after solidification, because the ionic radius of the high-valence cations in the curing agent was small, and it replaced the low-valence cations, which reduced the negative electric potential on the mineral surface, which thus reduced the ζ potential on the surface and the gap between the soil particles. Additionally, the properties of soil surface were changed by the corresponding ion exchange reaction. Because colloidal particles can invade the hydration film on the surface of clay minerals, the overall thickness of the water film can be effectively reduced, thus enhancing the bonding between the minerals in the soil.

In addition, it can be seen from the results of the pH test that the pH of the solidified soil slightly increased with increases in the curing age and curing agent content, which reflected that the curing process had a certain influence on the pH of the soil. By comparing the pH value of cement- and lime-solidified soil with age, we found that the curing agent had no great influence on the soil and will not have a great influence on the environment around the construction site.

## 4. Conclusions

(1) With the incorporation of a curing agent, some small cemented substances gradually appeared on the surface of soil minerals, which filled the gaps between the minerals well. At the same time, with the increase in the curing period, there were more colloidal particles in the soil, and some of them agglomerated into larger clump-like structures attached to the surface of soil granular minerals. It could be seen that with the incorporation of curing agent and the increase in its curing age, the cohesion and integrity between different particles enhanced, which produced an overall more compact soil structure.

(2) The curing period had a certain effect on the pH of the solidified soil, but this effect was not obvious. We found that the road liquid curing agent will not have a significant impact on the environment through a comparison with the pH of inorganic solidified soil such as lime.

(3) Compared with the content analysis of plain soil, no new chemical elements were produced in the solidified soil. However, with the incorporation of the curing agent, the minerals in the soil reacted with it to form a cemented substance, and more organic macromolecular chain substances were preserved in the soil. At the same time, the content of Al ions significantly increased, which indicated that the addition of curing agent effectively enhanced the cohesion between molecules to achieve a curing effect.

(4) The analysis and comparison of the functional groups of plain soil, curing agent, and cured soil showed that the curing agent in the road liquid polymer reacted with some mineral components in the solid soil and further formed relatively stable cemented substances that exist in clay minerals.

(5) There were no new minerals after curing process, the curing process changed the adsorption type of the soil surface ions, and high-valence cations were used to replace low-valence cations with large radii. Furthermore, the process changed the mineral surface charge size and zeta potential, thereby reducing the subcrystalline size of the minerals and increasing soil compactness.

(6) Combined with the above analysis and referring to the curing principle of organic curing agent and inorganic curing agent, we inferred that the liquid curing agent mainly participated in ion substitution and gelling reaction in the soil, which made the soil structure more solid and stable to a certain extent.

## Figures and Tables

**Table 1:** The cumulative percentage of particle gradation.

Bore Diameter/mm	10	5	2	1	0.5	0.25	0.075
Passing Rate/%	100	98.25	94.21	89.74	82.55	80.21	76.2

**Table 2:** Relationship between dry density and moisture content of plain soil.

Moisture Content (%)	13.7	15.8	17.8	19.6	21.5
Dry Density (g/cm^3^)	1.798	1.838	1.832	1.776	1.717

**Table 3:** Technical requirements of curing agent.

No.	Technical Index	Technical Requirements
1	Appearance	Uniform state, no precipitation or flocculence
2	Solids content (%)	≥40
3	Density (g/cm^3^)	Within 0.03 of designed values ±
4	pH	6.5~8.5
5	Solubility	Dissolve completely
6	Stability	Difference between solid content of upper and lower layers of 28 d is not more than 3%
7	Modified water-dispersed epoxy oligomer content(%)	≥5

**Table 4:** Comparative analysis of mineral subcrystalline size.

Mineral	Subcrystalline Size of Plain Soil/nm	Subcrystalline Size of Solidified Soil/nm	Rate of Change/% (Rate of Change/Plain Soil)
Quartz	15.92	10.59	50.35
Kaolinite	41.06	31.29	31.23
Smectite	7.75	5.43	42.71
Illite	22.56	18.11	24.57

## Data Availability

All data, models, and codes generated or used during the study appear in the submitted article.
